# Dropping out of a peripartum depression mHealth study: participants’ motives and suggestions for improvement

**DOI:** 10.1186/s12874-025-02462-z

**Published:** 2025-01-11

**Authors:** Hanna Wierenga, Konstantina V. Pagoni, Alkistis Skalkidou, Fotios C. Papadopoulos, Femke Geusens

**Affiliations:** 1 Department of Sociology, University of Tübingen, Tübingen, Germany; 2 Department of Women’s and Children’s Health – Obstetric & Reproductive Health Research, Uppsala University, Uppsala, 751 85 Sweden; 3 Department of Medical Sciences – Psychiatry, Uppsala University, Uppsala, Sweden; 4 Department of Development and Regeneration – REALIFE group, KU Leuven, Leuven, Belgium

**Keywords:** mHealth, Peripartum depression, Pregnancy apps, Research apps, Drop out analysis, User perspectives

## Abstract

**Background:**

Peripartum depression is a common but potentially debilitating pregnancy complication. Mobile applications can be used to collect data throughout the pregnancy and postpartum period to improve understanding of early risk indicators.

**Aim:**

This study aimed to improve understanding of why women drop out of a peripartum depression mHealth study, and how we can improve the app design.

**Method:**

Participants who dropped out of the Mom2B study (*n* = 134) answered closed and open questions on their motives for dropping out of the study, suggestions for improvement, and preferred timeframe of the study. A mix of quantitative and qualitative strategies was used to analyze the responses.

**Results:**

The most common reasons for discontinuation were lack of time, problems with or loss of the pregnancy, the use of other pregnancy applications, surveys being too lengthy, the app draining too much battery, and participants incorrectly believing that their answers were irrelevant for the study. Participants suggested fewer survey moments, more reminders, and a need for more unique content compared to commercially available apps.

**Conclusions:**

Researcher who want to use mHealth designs in peripartum studies need to ensure that their study designs are as time-efficient as possible, remind participants about the study, manage expectations about the study and what is expected of participants throughout the study, design their apps to be attractive in a competitive market, and follow-up with participants who are excluded from the study due to pregnancy complications.

Approximately 14% of women suffer from peripartum depression (PPD) during their pregnancy or in the postpartum period [1]. Two to three months after childbirth is a common time for the onset of PPD, but up to one in ten women start suffering from PPD already *during* their pregnancy [2, 3]. Considering the negative effects of PPD on the quality of life of mothers [4], as well as the health and development of their offspring [5], it is important to improve our understanding of early risk indicators, as early recognition and treatment improves outcomes [6].

One way to do this, is by collecting data via mobile phone health (mHealth) applications to get a full picture of factors influencing women’s mental health during and after pregnancy. Most childbearing parents already use pregnancy apps [7, 8]. This is not surprising, as phone-based pregnancy applications can meet health information needs [9], which are typically elevated during pregnancy [10]. Most women use pregnancy apps to monitor their mental and physical health as well as the growth of the baby during pregnancy [11]. In these applications women can, for example, access information, consult health care professionals or track changes in their health, such as mood shifts [11, 12]. Previous research has proposed that these mobile applications have the potential to improve pregnant women’s mental and physical wellbeing [12, 13]. In addition, mHealth tools have been shown to successfully aid in treating gestational diabetes [12], and to reduce pregnant and postpartum women’s depressive symptoms [14].

However, mHealth applications can not only be valuable to parents, but also be powerful tools for researchers to collect data. This can be accomplished by integrating surveys and other data collection methods, such as data from the mobile phone’s sensors, into the apps. This data collection, also called digital phenotyping, offers unique advantages in assessing the physical and mental health of the user almost in real time and in the user’s real-life environment [15]. An important but under-studied aspect of this kind of research is to assess what makes these mHealth research applications successful, in terms of acceptance and usage by the end-users. Current mHealth usability and design research typically focuses on disease management and health promotion applications, rather than data collection applications [16, 17]. Several reviews have been published on the challenges in engaging participants and reducing attrition in mHealth interventions [18, 19], indicating that reasons for dropping out of mHealth studies vary from application-related technical issues to individual issues such as a chaotic home environment or not feeling a need for the mHealth tool [20–22]. In addition, low user-friendliness and privacy concerns are important concerns [20]. Anonymity becomes especially important when data generated from the applications is used for research purposes and includes mental health related information [12, 20, 21]. With regard to peripartum mHealth tools, we also know that young women generally have a higher acceptance rate for mHealth tools [23]. Nevertheless, mHealth tools also suffer from high dropout rates and engaging mothers of newborns is challenging [22, 24].

These studies provide us with good insights in the challenges of designing mHealth applications for interventions to improve patient health, but it is not clear whether the same challenges exist for research apps designed for data collection, lacking an intervention component. Therefore, the aim of this study is to improve our understanding of why participants drop out of peripartum app-based data collection studies by investigating the dropout data of the Mom2B study. Mom2B is a Swedish PPD-mHealth project using a smartphone application in which women can answer surveys and provide data through active and passive methods, such as audio recordings and GPS tracking [25]. Mom2B is co-created by maternal health specialists, psychiatrists, psychologists, software engineers and researchers, to collect data throughout pregnancy and postpartum, ultimately aiming to contribute to the prediction and prevention of PPD [25].

There are three main objectives in this study. Firstly, we seek to further understand participants’ motives for discontinuing their use of a peripartum research app. Secondly, we aim to understand how participants would like to see such research tools improved. Finally, we aim to identify timepoints when pregnant and postpartum women prefer using research apps more intensively. This will provide crucial insights in how to improve research apps for data collection in peripartum mental health research.

## Methods

### Study design and recruitment

This is an exploratory study investigating participants’ decisions for dropping out of the Mom2b study (For the study’s protocol see: Bilal et al., 2022 [25]). Women over the age of 18 who are pregnant and fluent in Swedish can enroll in the Mom2B study. There were no other exclusion or inclusion criteria. In the research app, data are collected from the first week of pregnancy, up until one year after birth, and women can enroll any time during the pregnancy up to three months postpartum. Every week, participants receive two to three surveys with approximately five questions per survey. In addition, every 2–4 weeks women are prompted to follow specific instructions and make voice recordings. Passive data on mobility, internet usage, smartphone usage and social media are continuously collected if the participants have consented. Supplementary information is collected via the Swedish national health and quality registers for example to confirm that participants were pregnant and delivered a newborn during the study period.

**Fig. 1:**
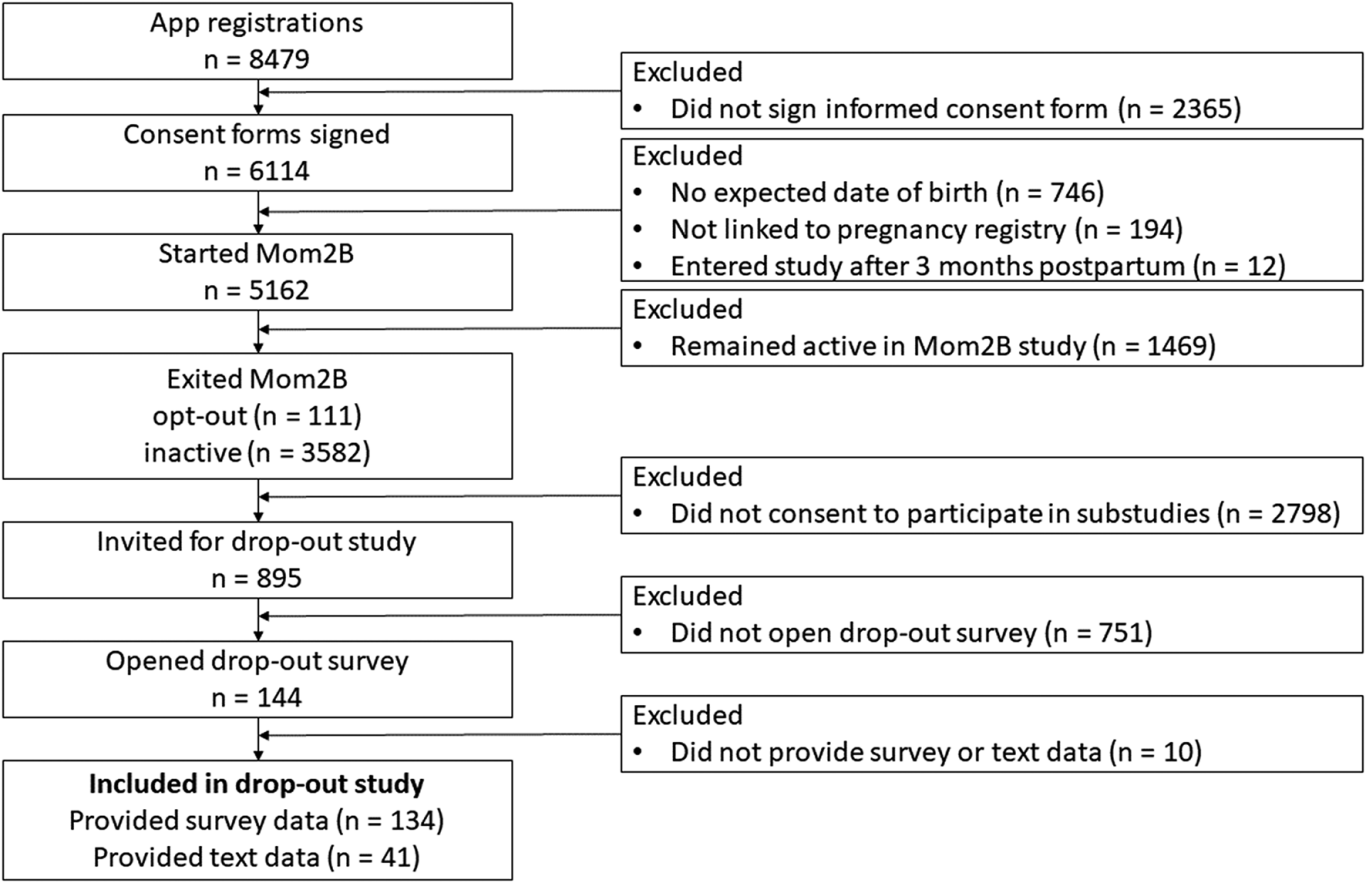
Flowchart on the inclusion and exclusion of the participants

In December 2022, 3 years after the original launch of the Mom2B research app, the study had recruited 6114 participants. For the present sub-study, participants who dropped out were recruited by sending out email invitations to previous participants who had dropped out of the study but had given consent to be contacted for sub-studies related to the main project (please see Fig. 1 for recruitment flowchart). This subsample served as the basis of our participants, who had dropped out of the study a month to three years prior to recruitment. On average, women used the Mom2B app for 144 days (M = 143.77, SD = 151.24) before dropping-out. Considering that, on average, they entered the study around day 146 (M = 146.40, SD = 82.79) of their pregnancy, participants typically dropped out around the birth of their child^1^. We defined dropout as participants who actively *exited* the study, or who did not contribute any data (surveys, voice recording and/or passive data) for a period of 6 months. Those who miscarried and consequently no longer met the inclusion criteria of the Mom2B research app were also included in this study if they had not communicated this to the research team. Participants did not need to declare why they dropped out of the study when they exited the study.

The questionnaire used in the current study was designed by the research team with the aim to explore motives behind the participants dropping out and improving app design. Utilizing a mixed methods approach, the survey included 3 questions in Swedish, both closed and open-ended, as well as a space for the participants to reflect on the app by providing free-text comments. The three questions related to participants’ motives for dropping out of the study, suggestions on how to improve the app, and which period of pregnancy or postpartum they deemed best to receive most surveys were included. The survey was anonymous and administered online through REDCap, a secure web-based application often used for the collection of data in clinical research [26]. No sociodemographic data was collected to minimize survey length and maximize response. The survey items and answer options were developed by the senior researchers (AS and FP), based on Mom2B specific features and previous literature [18, 27].

## Measures

**Motives for dropping out** Participants were provided with 11 potential reasons for dropping out of the study (see Table 1), and were asked to indicate whether this was a reason for them to discontinue their participation (= 1) or not (= 0). The potential reasons and participants’ answers are presented in Table 1. Participants were also provided with an open text field (named: *other*) where they could list other reasons for dropping out.

**Table 1:** Most common reasons for dropping out of the study

Most common reasons for dropping out of the study	*N*
1. Lack of time	49 (34,0%)
2. Problems with pregnancy	26 (18,1%)
3. Use of other pregnancy apps limits time for and usability of Mom2B	18 (12,5%)
4. Lengthy surveys	16 (11,1%)
5. App is not what I expected	12 (8,3%)
6. Many notifications	11 (7,6%)
7. Technical issues	9 (6,3%)
8. Surveys are difficult/unpleasant to answer	7 (4,9%)
9. Concerns about personal details/data	3 (2,1%)
10. Personal statistics are not always correct	1 (0,7%)
11. Other reason (allowing participants to use a free text field)	42 (29,2%)

**Suggestions for improvement** Participants were asked what could have been done better in the Mom2B app, and were provided with an open text field to answer.

**Ideal peripartum period for data collection.** Finally, participants were asked during which periods they recommend that we ask the most questions in the Mom2B app. They were given seven non-exclusive options to choose from (see Table 2).

**Table 2:** Common concepts for the discontinuance of participation based on the free text field

Concept		*N*	Example answer
* Participant relevance issues*			
	Loss of pregnancy (miscarriage or childbirth)	8	Miscarriage
	Was feeling well	4	I was feeling well the whole time and didn’t have any problems
	No longer pregnant but unknown what happened to child	2	I’m not pregnant anymore and felt I wasn’t contributing
	Feeling that a long time has passed since pregnancy	2	My child was born 18 months ago
	Unclear to participant that the study continued in the postpartum period	1	The child was born
	Feeling like an outlier due to emotional trauma	1	I experienced emotional trauma and my answers did not feel fair to the study.
* App issues*			
	Too battery heavy	4	Several features required the app to be running all the time/draw extra battery
	No reminders received	3	I have not received any more surveys. Willing to continue. Haven’t received a reminder either.
	Issues when switching phones	2	changed phone and then everything disappeared and had to redo everything
	Update issues	1	I gave birth and had the app a couple of weeks after birth and then the app did not update.
	Question layout inconsistent	1	the app was quite poorly designed I think and the answers came differently to different questions. For example, the answer Yes was at the top of one question and at the bottom of the next question. Was quite annoying when you knew immediately what you wanted to answer and couldn’t just choose the answer directly but had to “look” for the right answer.
*Survey issues*			
Too frequent		2	The questions should be answered so often - every week if I remember correctly? The frequent answering was the reason why I couldn’t take it anymore, especially after I had my baby.
	Unknown how long study lasts	1	nowhere stated how long the study would last
	Questions too intimate	1	The questions were a bit too intimate
	Wished-for answer options not included	1	my experiences were not represented in response options
	Too repetitive	1	Felt good throughout the pregnancy and then found it boring to answer the same thing all the time
* Miscellaneous*			
	Forgot about the app	3	Forgot to remove it/Forgot about it
	No time	1	have had children and do not have time to answer surveys
	Did not actively choose to stop	1	Have not actively chosen to exit
	Doesn’t know why stopped	1	don’t even know that I cancelled my participation

**Table 3:** Concepts around the improvement of the Mom2B app

Concept	*N*	Example answer
*Survey specific suggestions*		
Need for less repetition of questions and shorter surveys	14	very many questions too often, have fewer questions less often so you can continue answering for a longer period
Some questions or tasks are too difficult or intimate	4	Don´t ask such intimate questions
Specify study and survey length	3	I would have liked to see how many questions there were in each survey so you knew how long it would take approximately, sometimes there were a lot of questions and sometimes very few, you never knew when it would end.
Need for norm critical perspective in the surveys	1	more clearly incorporate norm-critical perspectives
*App specific suggestions*		
Need for more notifications	6	More reminders
Design system for data transfer between phones when reinstalling	3	Technology. Ended because when I switched phones my data was not transferable and I had to start over.
Need for the app to be more user-friendly	2	Make the app more user-friendly
Need for an offline version of the app	2	Fully offline would have been good
Add module on mental and physical health after miscarriage when guided out of the study	1	The app told me to delete it due to terminated pregnancy. But I was wondering if it would have been important in the study to follow the feeling when you are forced (i.e. aborted because the fetus would not have survived anyway) to abort or have a miscarriage. It would have been good to get questions and information from the app about what happens in the body after abortion and when you can physically think about trying again. Personally, it was a confusing period and felt difficult to find information about the process beyond the sheet you got from the hospital.
*Other*		
Positive or neutral comments	11	Thought they were both relevant and good questions.
Need for more information and unique content, including personal statistics	5	More information about each week and trimester, more information about the time after. What is normal to feel and when to seek care? Where to seek care, etc.
Clearly state that all responses are valuable	2	I think the biggest reason why I didn’t stay active was precisely that I’m bad at regularity, and felt that irregular activity might interfere with the study rather than help it.
Need for direct communication with the research team	1	Sent you an email in August with some concerns about the app and consequences after the birth, but have not yet received a reply, feel I don’t really have confidence in you after that.

### Analysis

The analysis of the quantitative data included descriptive statistics, more specifically frequencies, and was conducted using IBM SPSS Statistics (Version 28). In order to analyse the open-ended questions that complemented our quantitative data, we used conceptual content analysis which allowed us to explore the occurrence of specific answers in our dataset [28]. As this is an exploratory study, we used an inductive approach during the analysis of the qualitative data, which allowed us to observe patterns and themes and generate new theories emerging from our data [29]. The qualitative data was translated from Swedish to English and back before analysis by a Swedish speaking researcher (KVP), and the answers were analyzed in English. FG, HW and KVP independently coded the data, after which they discussed their codes. In case of differences, the researchers discussed the answer until consensus was reached.

### Ethical considerations

Approval for the current study had been obtained by the Swedish Ethical Review Authority (initial application dnr: 2019/01170, amendment dnr: 2021/04894). All participants had provided written consent during their initial registration for the Mom2B study to be contacted in case of further sub-studies, such as the current study and they were informed about its aims before completing the survey.

## Results

Out of the 895 invitations, 144 participants opened the survey; 134 of them filled out the questionnaire and 41 contributed with written text. See Fig. 1 for a flowchart of the inclusion of the participants.

### Reasons for discontinuing the participation

**Fig. 2:**
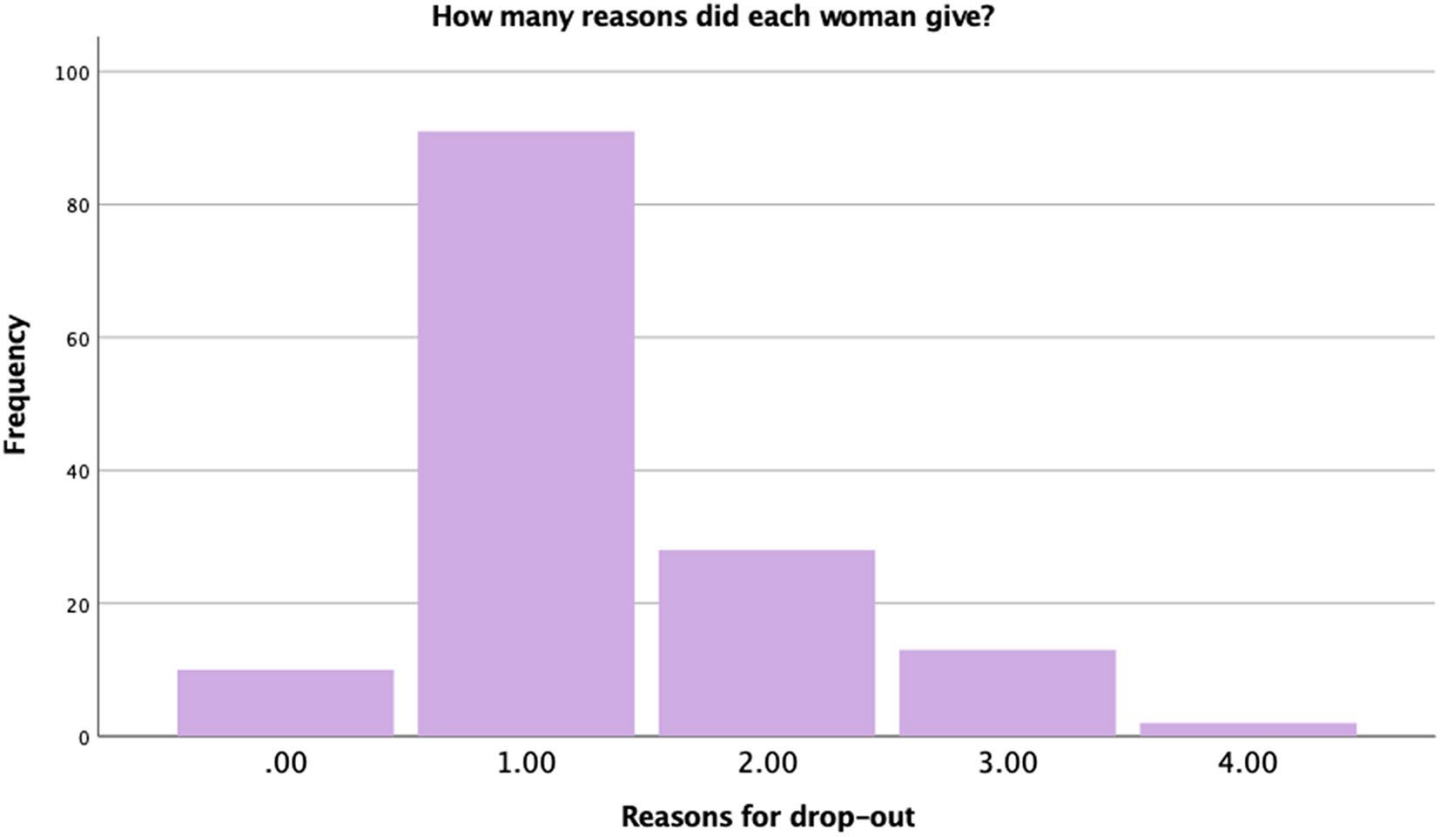
Number of reasons provided for dropping out

The frequencies of the different reasons for dropping out of the study are presented in Table 1. The most common reasons for discontinuation, among those provided in our list, was lack of time (34%), problems with pregnancy (18%), the use of other pregnancy applications instead of Mom2B, and that they perceived the surveys as too lengthy (11%). The reported number of different reasons for discontinuation is presented in Fig. 2, with most participants reporting only one reason for discontinuing their participation.

**Fig. 3:**
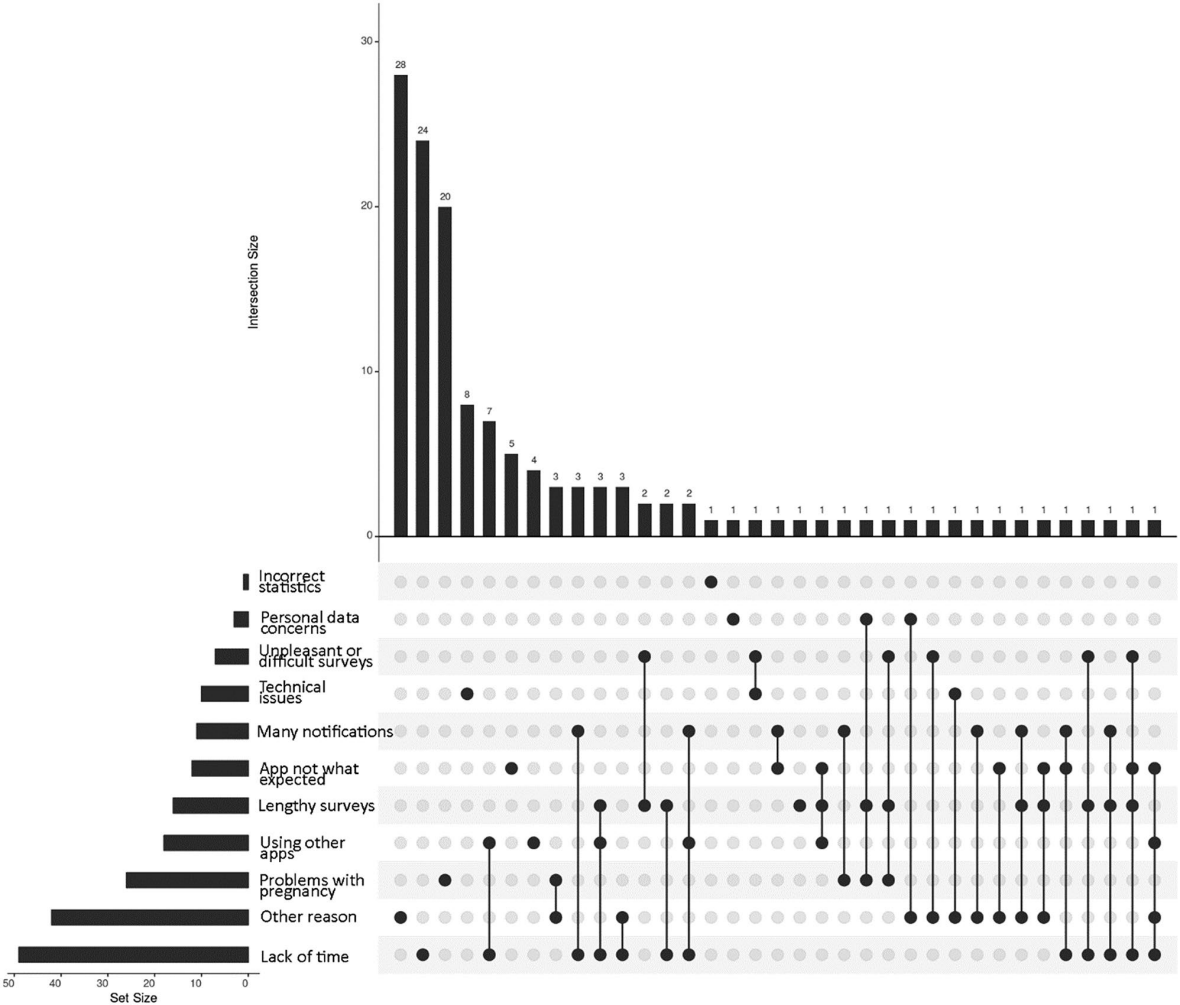
Interrelatedness between the different motives for dropping out of the study

Figure 3 shows the interrelatedness between the different answer options. Overall, this showed a clustering of a lack of time, lengthy surveys, the use of other pregnancy apps, and the app not being what expected. However, due to the limited sample size, no conclusive conclusions can be drawn on answer patterns.

One in three (29%) of the participants chose an “Other reason” for discontinuation. Upon selecting this alternative, a new box opened up for the participants so that they could elaborate on the details as free text. These free text answers were analyzed and the concepts that emerged during the content analysis are summarized in Table 3. While the answers here were often in line with the provided answer options, they were usually more nuanced or drew attention to specific or personal issues. This signaled that the participants had the need to specify their personal experience further. The most important reasons pointed out by the participants were the loss of a pregnancy due to miscarriage or stillbirth, the app draining too much battery throughout the day, and women believing that their answers were irrelevant or would skew the data. It should be noted that pregnancy loss was an exclusion criterion in the Mom2B study, so these women did not ‘drop out’ as such, but were excluded from further participation by the study design.

### Users suggestions for improvement

During the last open-ended question of the survey, 41 participants contributed their suggestions for the improvement of the Mom2B app. The main concepts for improvement are presented in Table 2 as well as how often each concept appeared in the data. While the surveys being too repetitive was reported as a motive for dropping out by only one participant, it was the most frequent suggestion for improvement. In addition, women wished for more notifications to remind them to fill out surveys. Many positive or neutral comments about the app were also written, indicating that a follow-up was appreciated. The positive comments were, for example, that the app was generally nice to use, the questions were relevant to their pregnancy-experience, and they were happy that these kind of studies were being conducted.

### Ideal peripartum period for data collection

Finally, when it came to the peripartum period during which women preferred to complete the most surveys, it is not clear which peripartum period women prefer. Most women chose multiple periods, indicating no clear preference for one period over another. However, as seen in Table 4, there was a tendency towards the pregnancy period, as well as the first three months postpartum.

**Table 4:** Preferred peripartum period for data collection

During which periods do you recommend that we ask the most questions in the Mom2B app?	*N*
1. Week 6–12 of pregnancy	57 (39,6%)
2. Week 12–25 of pregnancy	63 (43,8%)
3. Week 25–32 of pregnancy	56 (38,9%)
4. Week 32–40 of pregnancy	59 (41%)
5. Month 1–3 after giving birth	50 (34,7%)
6. Month 3–6 after giving birth	30 (20,8%)
7. Month 6–12 after giving birth	27 (18,8%)

## Discussion

By analyzing why participants dropped out of the Mom2B mHealth research project on PPD, this study aimed to provide insights in how to improve app design of data collection tools in peripartum mental health research. Based on our results, we can draw five major conclusions.

First, pregnancy and the postpartum period are intense periods in life, with parents having limited time resources while preparing to bring new life into this world or adjusting to their new roles as parents after the child is born. While the time-flexible nature of mHealth tools is a key advantage in this type of research, it is important to consider the time constraints and family-related demands faced by this demographic, who may use the app while stressed or in a hurry. The results from this study show that women find it difficult to make time for participation in research, especially time-intensive studies that require a prolonged engagement. Accommodating data collection designs may be important to reach this group, for example by reducing the number of questions in surveys to the least amount needed to answer the research questions or by focusing on the pregnancy period rather than the postpartum period. Participants emphasized the importance of not overburdening them with too many repetitive questions, a suggestion in line with recommendations for designing experience sampling studies [30]. We recommend that mHealth research apps for data collection sharpen their focus and avoid adding many subprojects and questions to their designs. This could potentially also help with the battery drainage issues, as fewer app-functions (e.g., no GPS tracking) could save battery life.

Second, based on the answers on both open questions, it is important to set reminders for participants to follow-up on their commitment. Not receiving reminders was an important reason for participants to drop out of our study, as this resulted in them forgetting about the app and the study. Following-up with participants and sending them frequent reminders to fill out surveys is a common retention strategy in longitudinal cohort studies, though its effectiveness is contested [31]. However, our results indicate that this could be a useful strategy for highly motivated participants who want to remain involved, but who may forget about the study without a reminder. Though this was not reflected on by our participants, frequent reminders at different times throughout the day may also be helpful for participants to plan their app engagement outside of the evening hours after work, when they may want to relax and connect with their partner, rather than answer survey questions about the hassles and stressors they have been dealing with all day. Reminders may not be as useful for participants who were planning on discontinuing their participation in the study for other reasons.

Third, we need to set clear expectations about what the study entails, what is expected of the participants or who the target population is. Several women reported feeling mentally well and they dropped out of the study because they were unsure of whether they would skew the data. Clarifying that all answers are valuable as well is important to reduce bias from high dropout rates from mentally healthy women or those who have experienced trauma or other adversities. In addition, we need to manage expectations about the study design, as many women were uncertain about when the study ended, how long they should continue using the app and how long the surveys would take to fill out. Additional information when reaching important milestones (e.g., at birth) about how much longer the study runs, may be helpful to remind participants of the design. Participants also suggested to provide an estimation of how long a specific survey will take to fill out, which could help them plan their day and may further improve retention. However, our findings also show that providing participant information does not necessarily mean ‘informed’ consent. All of this information – with the exception of how long each specific survey takes to fill out - was provided to the participants at the start of the study, but they either did not clearly read the participant information form before signing the informed consent form, or they forgot as the study ran. Providing alternative options of receiving the participant information (e.g., video based instead of text based, having a research assistant go over the documents with them) and having a FAQ with all of the participant information in bite-sized portions within the app can help better inform participants and consequently help manage expectations.

Fourth, our data show that we are competing with commercial competitors providing pregnancy apps without research components. There is a wide range of pregnancy apps available for women to choose from, many of which receive good ratings in the available app stores [32]. One in ten of our respondents explicitly stated that their use of other pregnancy apps was the reason for dropping out of our study, and prior research on mHealth intervention apps has indicated that unsatisfactory functionality of research apps may hinder user engagement [33]. Additionally, several individuals suggested to add more unique content to our app to set it apart from other apps, or to provide more immediate benefits to the users. Translating research results into understandable output for users, such as average scores on important variables, or trends among app users, may help set out the research app compared to commercial competitors. Collaborating with researchers with a marketing and/or usability design background may be especially important to identify and meet the needs of app users and help users choose the research app over commercially available apps.

Finally, and unique to the pregnancy situation, is the need for more attention to pregnancy complications, in particular pregnancy loss. Pregnancy loss was one of the most important reasons for participants to exit the study, and women indicated that this was a crucially important moment for them to receive more information and follow-up, which was not included in the research app. Pregnancy loss and other pregnancy complications are often exclusion criteria for peripartum studies, but providing these individuals with additional information and support may be beneficial for their mental health, and to build rapport. Even if they are no longer included in the study, it could be helpful to provide them with reliable information, as well as with contact information for support groups or healthcare professionals who can aid them further. For example, women who report clinical PPD levels in our study receive a personal phone call to follow-up with them; this may also be beneficial for women who drop out of the study because of pregnancy complications or pregnancy loss.

### Limitations

Before finishing, we would like to note that our study focused on the reasons for discontinuing engagement with app-based data collection tools in peripartum mental health research. As such, we cannot make any statements on the quality of the data collected via such tools. It is possible that individuals continued using the app, but had ‘mentally dropped out’ of the study because of fatigue, boredom, or wanting to get over with the study without actively discontinuing their participation. Disengaged survey responses are a general issue that can occur in any study [34], so it could be useful to combine our app-based approach with an occasional in-person check-in from a researcher to increase participants’ engagement with the study and monitor the quality of the collected data. Furthermore, a more accessible in-person contact could facilitate better information delivery, resolving misconceptions about the participation and identifying women in need of support. In our study, participants were informed that they could email the research team with questions, but being available on chat or for a short call could lower the threshold for initiating contact.

Although our study provides unique insights into how we can improve the development of mHealth applications for research, there are some limitations to keep in mind as we interpret the results. First, no sociodemographic data were collected. As such, we cannot draw any conclusions on whether certain motivations for dropping out were more important for certain sociodemographic groups than others. It would have been insightful to test whether certain trends in attrition are linked to specific sociodemographic variables. Second, the study launched three years before participants were contacted, so it is possible that participants who dropped out of the study in the earlier stages have different recall of their motives for dropping out of the study than participants who dropped out of the study more recently. Recall bias is likely a bigger issue for participants who dropped out at the start of the study than participants who had more recently dropped out. Third, this study was conducted in Sweden, a country with excellent maternal healthcare covered by universal health insurance. As such, Swedish women may have less need to rely on apps for reliable information, as they have easy access to midwives. In addition, our Swedish participants also generally did not report any concerns about their privacy or data management, a concern which may be more prominent in countries with less strict data management laws. For example, there is some worry in the USA about pregnancy or menstrual tracking apps sharing sensitive data in the post Roe v. Wade era [35]. It is possible that app use in Sweden differs from app use in other countries without socialized healthcare, safe access to abortions or strict data management laws, potentially resulting in different motivations to drop out of peripartum mHealth studies. Lastly, we would like to highlight that participants might drop out if they have misconceptions or stigmatized beliefs about mental health, which was not assessed in our survey.

## Conclusion

mHealth research applications can be useful tools to collect research data. However, a careful analysis of motivations to discontinue research app use is important to improve app design and study results. Based on the dropout analysis of the Mom2B data collection app to predict and prevent PPD, we conclude that researchers who want to use mHealth designs need to (1) make their study designs as time-efficient as possible, (2) ensure that participants receive reminders, (3) manage expectations about the study and what is expected of participants, (4) remember that they are competing with many commercial competitors, and (5) provide follow-up for participants who are excluded from the study due to pregnancy complications.

## Data Availability

Data are available upon reasonable request from AS.

## Footnotes

1 Note: this is based on the full Mom2B sample.

## References

[1] Liu X, Wang S, Wang G. Prevalence and risk factors of Postpartum Depression in women: a systematic review and Meta-analysis. J Clin Nurs. 2022;31:2665–77. 34750904 10.1111/jocn.16121

[2] Wikman A, Axfors C, Iliadis SI, Cox J, Fransson E, Skalkidou A. Characteristics of women with different perinatal depression trajectories. J Neurosci Res. 2020;98:1268–82. 30723972 10.1002/jnr.24390

[3] Woody CA, Ferrari AJ, Siskind DJ, Whiteford HA, Harris MG. A systematic review and meta-regression of the prevalence and incidence of perinatal depression. J Affect Disord. 2017;219:86–92. 28531848 10.1016/j.jad.2017.05.003

[4] Li J, Yin J, Waqas A, Huang Z, Zhang H, Chen M, et al. Quality of life in mothers with perinatal depression: a systematic review and meta-analysis. Front Psychiatry. 2022;13:734836. 35242060 10.3389/fpsyt.2022.734836 PMC8886107

[5] Slomian J, Honvo G, Emonts P, Reginster J-Y, Bruyère O. Consequences of maternal postpartum depression: a systematic review of maternal and infant outcomes. Womens Health. 2019;15:174550651984404. 10.1177/1745506519844044 PMC6492376 31035856

[6] Howard MM, Mehta ND, Powrie R. Peripartum depression: early recognition improves outcomes. Cleve Clin J Med. 2017;84:388–96. 28530897 10.3949/ccjm.84a.14060

[7] Lupton D, Pedersen S. An Australian survey of women’s use of pregnancy and parenting apps. Women Birth. 2016;29:368–75. 26874938 10.1016/j.wombi.2016.01.008

[8] Lanssens D, Thijs IM, Dreesen P, Van Hecke A, Coorevits P, Gaethofs G, et al. Information resources among flemish pregnant women: cross-sectional study. JMIR Form Res. 2022;6:e37866. 36222794 10.2196/37866 PMC9597425

[9] Wang N, Deng Z, Wen LM, Ding Y, He G. Understanding the use of Smartphone Apps for Health Information among pregnant Chinese women: mixed methods study. JMIR MHealth UHealth. 2019;7:e12631. 31215516 10.2196/12631 PMC6604500

[10] Ghiasi A. Health information needs, sources of information, and barriers to accessing health information among pregnant women: a systematic review of research. J Matern Fetal Neonatal Med. 2021;34:1320–30. 31216921 10.1080/14767058.2019.1634685

[11] Doherty K, Barry M, Marcano-Belisario J, Arnaud B, Morrison C, Car J, et al. A Mobile App for the self-report of Psychological Well-being during pregnancy (BrightSelf): qualitative design study. JMIR Ment Health. 2018;5:e10007. 30482742 10.2196/10007 PMC6290271

[12] van den Heuvel JF, Groenhof TK, Veerbeek JH, van Solinge WW, Lely AT, Franx A, et al. eHealth as the Next-Generation Perinatal Care: an overview of the literature. J Med Internet Res. 2018;20:e202. 29871855 10.2196/jmir.9262 PMC6008510

[13] Hantsoo L, Criniti S, Khan A, Moseley M, Kincler N, Faherty LJ, et al. A Mobile Application for Monitoring and Management of Depressed Mood in a vulnerable pregnant Population. Psychiatr Serv. 2018;69:104–7. 29032705 10.1176/appi.ps.201600582 PMC5750085

[14] Lau Y, Chew HSJ, Ang WHD, Ang WW, Yeo CY, Lim GZQ et al. Effects of digital health interventions on the psychological outcomes of perinatal women: umbrella review of systematic reviews and meta-analyses. Health Psychol Rev. 2023;:1–26. 10.1080/17437199.2023.2185654 36919443

[15] De Boer C, Ghomrawi H, Zeineddin S, Linton S, Kwon S, Abdullah F. A call to expand the scope of digital phenotyping. J Med Internet Res. 2023;25:e39546. 36917148 10.2196/39546 PMC10132029

[16] Noorbergen TJ, Adam MTP, Roxburgh M, Teubner T. Co-design in mHealth Systems Development: insights from a systematic literature review. AIS Trans Hum-Comput Interact. 2021;13:175–205.

[17] Keusch F, Bähr S, Haas G-C, Kreuter F, Trappmann M, Eckman S. Non-participation in smartphone data collection using research apps. J R Stat Soc Ser Stat Soc. 2022;185 Supplement2:S225–45.

[18] Amagai S, Pila S, Kaat AJ, Nowinski CJ, Gershon RC. Challenges in participant engagement and retention using mobile health apps: literature review. J Med Internet Res. 2022;24:e35120. 35471414 10.2196/35120 PMC9092233

[19] Meyerowitz-Katz G, Ravi S, Arnolda L, Feng X, Maberly G, Astell-Burt T. Rates of attrition and dropout in app-based interventions for chronic disease: systematic review and meta-analysis. J Med Internet Res. 2020;22:e20283. 32990635 10.2196/20283 PMC7556375

[20] Ben-Zeev D, Schueller SM, Begale M, Duffecy J, Kane JM, Mohr DC. Strategies for mHealth Research: lessons from 3 mobile intervention studies. Adm Policy Ment Health Ment Health Serv Res. 2015;42:157–67. 10.1007/s10488-014-0556-2 PMC4232479 24824311

[21] Attard R, Iles J, Satherley R-M. How acceptable do parents experiencing mental health challenges find e-Health interventions for mental health in the postnatal period: a systematic review. BMC Pregnancy Childbirth. 2022;22:763. 36224526 10.1186/s12884-022-05070-7 PMC9554391

[22] Pinto-Foltz MD, Logsdon MC, Derrick A. Engaging adolescent mothers in a longitudinal Mental Health intervention study: challenges and lessons learned. Issues Ment Health Nurs. 2011;32:214–9. 21355755 10.3109/01612840.2010.544841 PMC3079417

[23] Kenter R, Warmerdam L, Brouwer-Dudokdewit C, Cuijpers P, van Straten A. Guided online treatment in routine mental health care: an observational study on uptake, drop-out and effects. BMC Psychiatry. 2013;13:43. 23368894 10.1186/1471-244X-13-43 PMC3577663

[24] Torous J, Lipschitz J, Ng M, Firth J. Dropout rates in clinical trials of smartphone apps for depressive symptoms: a systematic review and meta-analysis. J Affect Disord. 2020;263:413–9. 31969272 10.1016/j.jad.2019.11.167

[25] Bilal AM, Fransson E, Bränn E, Eriksson A, Zhong M, Gidén K, et al. Predicting perinatal health outcomes using smartphone-based digital phenotyping and machine learning in a prospective Swedish cohort (Mom2B): study protocol. BMJ Open. 2022;12:e059033. 35477874 10.1136/bmjopen-2021-059033 PMC9047888

[26] Patridge EF, Bardyn TP. Research Electronic Data Capture (REDCap). J Med Libr Assoc JMLA. 2018;106:142–4.

[27] Conway M. Determining the role of the internet in violent extremism and terrorism: six suggestions for progressing research. Stud Confl Terror. 2017;40:77–98.

[28] Krippendorff K. Content analysis: An introduction to its methodology. 2455 Teller Road, Thousand Oaks California 91320: SAGE Publications, Inc.; 2019.

[29] Thomas DR. A general inductive approach for analyzing qualitative evaluation data. Am J Eval. 2006;27:237–46.

[30] Scollon CN, Kim-Prieto C, Scollon CN. Experience sampling: promises and pitfalls, strengths and weaknesses. J Happiness Stud. 2003;4:5–34.

[31] the SEED Lifecourse Sciences Theme, Teague S, Youssef GJ, Macdonald JA, Sciberras E, Shatte A, et al. Retention strategies in longitudinal cohort studies: a systematic review and meta-analysis. BMC Med Res Methodol. 2018;18:151. 30477443 10.1186/s12874-018-0586-7 PMC6258319

[32] Musgrave LM, Kizirian NV, Homer CSE, Gordon A. Mobile phone apps in Australia for improving pregnancy outcomes: systematic search on App stores. JMIR MHealth UHealth. 2020;8:e22340. 33196454 10.2196/22340 PMC7704277

[33] Koh J, Tng GYQ, Hartanto A. Potential and pitfalls of mobile mental health apps in traditional treatment: an umbrella review. J Pers Med. 2022;12:1376. 36143161 10.3390/jpm12091376 PMC9505389

[34] Soland J, Wise SL, Gao L. Identifying disengaged survey responses: new evidence using response time metadata. Appl Meas Educ. 2019;32:151–65.

[35] Dong Z, Wang L, Xie H, Xu G, Wang H. Privacy Analysis of Period Tracking Mobile Apps in the Post-Roe v. Wade Era. In: Proceedings of the 37th IEEE/ACM International Conference on Automated Software Engineering. Rochester MI USA: ACM; 2022. pp. 1–6.

